# miR-129-5p Inhibits Adipogenesis through Autophagy and May Be a Potential Biomarker for Obesity

**DOI:** 10.1155/2019/5069578

**Published:** 2019-11-06

**Authors:** Xue Fu, Lina Jin, Luyu Han, Yini Yuan, Qian Mu, Hui Wang, Jian Yang, Guang Ning, Donglei Zhou, Zhiguo Zhang

**Affiliations:** ^1^Department of Endocrinology and Metabolism, Shanghai Institute of Endocrine and Metabolic Diseases, Ruijin Hospital, Shanghai Jiao Tong University School of Medicine, Shanghai, China; ^2^Department of Gastrointestinal Surgery, Shanghai Tenth People's Hospital Affiliated to Tongji University, Shanghai, China

## Abstract

**Introduction:**

Obesity has an unclear pathogenesis. MicroRNAs (miRNAs) may function as biologically active molecules for obesity through regulating adipocyte differentiation. This study aimed to identify how miR-129-5p (a specific miRNA) regulates adipogenesis in vitro and explore its possible role in the pathogenesis of obesity in humans.

**Materials and Methods:**

The miR-129-5p expression was detected in obese mouse models. The effect of miR-129-5p on adipocyte differentiation was observed, and the adipose markers were analyzed. Bioinformatics and dual-luciferase reporter assay were applied to predict and confirm the target genes of miR-129-5p. The human serum samples were detected and analyzed.

**Results:**

miR-129-5p is highly expressed in adipose tissues of *db/db* mice. Gain- and loss-of-function studies show that miR-129-5p could significantly inhibit adipocyte differentiation and white adipocyte browning in vitro and decreases the level of specific markers, such as FABP4, UCP1, and PPAR*γ*, in mature white and brown adipocytes. miR-129-5p directly targets ATG7 which is predicted with bioinformatics and confirmed by dual-luciferase reporter assay. Serum miR-129-5p level was evidently elevated in patients with simple obesity (*p* < 0.01) and correlates with obesity indices, including BMI (*r* = 0.407, *p* < 0.029) and fat percentage (*r* = 0.394, *p* < 0.038).

**Conclusion:**

miR-129-5p might target on the ATG7-related autophagy signaling network that regulates white and brown adipogenesis. Importantly, the aforementioned results suggest serum miR-129-5p might be a potential biomarker and therapeutic target for obesity.

## 1. Introduction

Obesity is an epidemic health problem worldwide and a major contributor to metabolic syndrome and disorders, such as type II diabetes, nonalcoholic fatty liver disease, cardiovascular disease, and some cancers [1–3].

Obesity is defined as excessive fat accumulation in adipose tissue [4]. Mammals have three types of adipocytes, white, classical brown, and beige adipocytes. White adipocytes specialize in energy storage, while brown adipocytes specialize in energy expenditure without generating ATP. In addition to the classical brown adipocytes, beige adipocytes represent UCP1-expressing brown adipocytes emerging in white adipose tissue upon certain stimulations [5, 6].

MicroRNAs (miRNAs) are a novel group of small (approximately 22 nucleotides) noncoding RNAs that emerge as important regulators of mRNA expression [7]. Increasing evidence has demonstrated that plenty of miRNAs have function on obesity through regulating adipogenesis [8]. Adipogenesis is a complex process and contains two main stages, commitment and differentiation. Once preadipocytes (or stem cells) commit to an adipose lineage, they are induced to form mature adipocytes requiring sequential activation of transcription factors, including CCAAT/enhancer-binding protein (C/EBP) gene family and peroxisome proliferator-activated receptor-*γ* (PPAR*γ*) [9]. miRNAs have been reported to modulate adipocyte differentiation by targeting adipogenic regulators. For example, miR-143 enhances the differentiation of cultured human preadipocytes and directly targets FGF7 which may function as a fine-tuning molecule in the adipogenic process [10]. Similarly, miR-27b directly targets PPAR*γ* and inhibits the process of human adipogenesis [11]. The evidence suggests that different miRNAs have different effects on adipocyte differentiation, and which adipocyte-specific genes are regulated by specific miRNA is not clear so far. Furthermore, with the advancement of technology, circulating miRNAs are treated as potential biomarkers for obesity. For example, miR-223, miR15b, and miR130b increase in individuals and overweight people with obesity [12]. However, it remains unclear whether adipocyte-functioned miRNAs will become novel biomarkers for obesity.

In this work, the regulating functions of a specific miRNA in adipogenic program were investigated. Based on our study, we analyzed and confirmed the direct target genes of miR-129-5p in vitro and determined the possible signaling pathway mediating adipocyte differentiation and the browning program of white adipocytes. Moreover, we explored the associations between circulating miR-129-5p and parameters of obesity and aimed to provide novel therapeutic targets for defeating obesity.

## 2. Materials and Methods

### 2.1. Animal Experiments

This animal study was approved by the Animal Care Committee of Shanghai Jiao Tong University School of Medicine. The male *db*/*db* mice generated in C57BLKS/J background and wild-type littermates were purchased from the Model Animal Research Center of Nanjing University (Nanjing, China, Approval No. SCXK (SU) 2015-0001). 7-week mice were housed at a 12-hour light/dark cycle with free access to water and food. After 1-week adaptation, the mice were sacrificed for subsequent experiments.

### 2.2. Isolation of SVF Cells

The C57BL/6 genetic background mice were purchased from Lingchang Biotech, China. Primary white fat stromal vascular and mature fat cells were fractionated according to published methods [13, 14]. Then, cell culture and adipocyte differentiation were established as previously described [15].

### 2.3. HEK 293T Cell Culture

Human embryonic kidney (HEK) 293T cells (ATCC, Manassas, VA) were cultured in Dulbecco's modified Eagle's medium (DMEM) (Hyclone, Logan, UT) supplemented with 10% fetal bovine serum (FBS) (Hyclone), 100 units/ml penicillin, and 100 mg/ml streptomycin (Invitrogen, Carlsbad, CA, USA) and maintained in 5% CO_2_ at 37°C [16].

### 2.4. Prediction of miRNA Targets and Bioinformatic Analysis

Target genes were predicted by TargetScan (http://www.targetscan.org/) and miRDB (http://www.mirdb.org/). The web-based computational tool DIANA Lab (http://www.microrna.gr/miRPathv2, accessed July 2012) was used to identify signaling pathways potentially altered by miR-129-5p targets.

### 2.5. Dual-Luciferase Reporter Assay

HEK 293T cells were cotransfected with Luc-3′UTR constructs and a control mimic or a miR-129-5p mimic (Ribobio, Guangzhou, China). Luciferase activities were measured using Dual-Luciferase Kit for Luc-ATG7, HMGB1, INSIG1, SOX2, and TMEM65-3′UTR following the manufacturer's protocols (Promega, USA).

### 2.6. miRNA and RNA Analysis

Serum miRNA analysis was performed as previously described [17]. RNA extraction, cDNA synthesis of genes, and real-time quantitative PCR (RT-qPCR) were also performed as previously described [18]. Briefly, total RNAs were first dissolved using QIAzol reagent (Qiagen, Germany) according to the manufacturer's instructions and then subjected to standard total miRNA extraction and cDNA synthesis of fat tissue and cells. For RT-qPCR analysis, *C*_t_ values <34 were used for gene expression analysis. Primers are presented in Table 1. Ce_miR-39, U6, and *β*-ACTIN were used as internal controls for normalization for RT-qPCR of serum, tissue, and cell miRNAs and protein-coding genes, respectively. The sequences of miR-129-5p were synthesized by Qiagen, Germany. All the RT-qPCR results were expressed as a ratio in arbitrary units.

### 2.7. Western Blotting

Cells were lysed with RIPA buffer (Biocolors Biology, Shanghai, China). The protein concentration was assayed using a Pierce BCA Protein Assay Kit (Thermo Scientific, USA). The boiled samples were separated by SDS-PAGE and electrotransferred to PVDF membranes (Millipore, USA). The immunoblots were blocked with 10% nonfat milk and incubated with antiantibodies for UCP1, *β*-ACTIN (Santa Cruz, USA), PGC-1*α*, PPAR*γ*, FABP4, and FAS (Cell Signaling Technology, USA) overnight at 4°C. After that, the membranes were incubated with horseradish peroxidase-conjugated secondary antibodies (Cell Signaling Technology, USA), and target protein bands were detected using an enhanced chemiluminescence system (Millipore, USA). Anti-*β*-ACTIN antibodies were employed as internal control total cellular proteins.

### 2.8. Oil Red O Staining

At day 6 or day 8 of differentiation, adipocytes were washed twice with phosphate-buffered saline (PBS) and stained with filtered Oil Red O solution (Nanjing Jiancheng Bioengineering Institute, China) for 15 min at room temperature according to the provided protocol of previous study [19]. Cells were visualized by light microscopy (Tokyo, Japan) and photographed to measure total lipid accumulation.

### 2.9. Measurement of Cell Triglyceride Levels

Cells were washed twice with l ml PBS on day 8 of differentiation and then dissolved in 220 *μ*l TG lysis buffer by sonication. The intracellular TG content was measured using a TG assay kit (Sigma-Aldrich, USA) according to the manufacturer's recommended protocol. Protein concentrations were quantified using a Pierce BCA Protein Assay kit (Thermo Fisher Scientific, USA). The results were expressed as milligram TG per milligram protein (mg TG/mg protein) and were standardized by dividing with the mimic control.

### 2.10. Study Subjects and Sample Collection

A total of 31 subjects were selected, including 15 normal weight volunteers from the Shanghai Jiao Tong University School of Medicine and 16 subjects with simple obesity from Ruijin Hospital. Clinical and biochemical measurements are shown in Table 2. The study was reviewed and approved by the Institutional Review Board of Ruijin Hospital, Shanghai Jiao Tong University School of Medicine, and was in accordance with the principle of the Helsinki Declaration II. All of the participants provided written informed consents. The fasting serum samples were collected from the participants and stored at −80°C.

### 2.11. Statistical Analysis

Each experiment was performed at least three times. Student's *t*-test was used to analyze the results of all cellular and animal experiments, and the results were presented as mean ± SEM. In the human trials, all values were presented as mean ± SD. Statistical analysis was performed using Student's *t*-test in SPSS 10.0 (SPSS Inc., Chicago, IL, USA). Pearson's correlation analysis was performed to examine the association between metabolic parameters and serum miR-129-5p. *p* < 0.05 was considered as statistically significant.

## 3. Results

### 3.1. miR-129-5p Levels Were Increased in Adipose Tissue of *db/db* Mice

**Figure 1:**
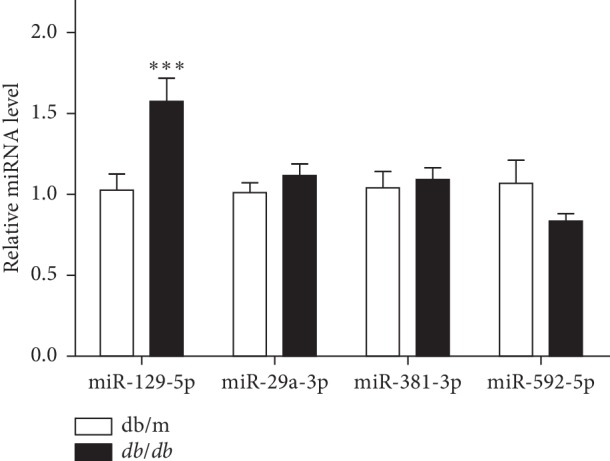
miR-129-5p level was highly expressed in EWAT of *db/db* mice. Relative expression level of different miRNAs in EWAT of *db/db* mice compared with wild-type mice (WT); *n* = 10 and ^*∗∗∗*^*p* < 0.001 compared with WT.

Based on previous research by our laboratory [17], we attempted to identify more miRNAs related to obesity. We screened differently expressed miRNAs in adipose tissues of obese mouse models and found that the miR-129-5p level was increased dramatically in epididymal white adipose tissue (EWAT) of *db/db* mice compared with the wild-type group (Figure 1). This finding indicates that miR-129-5p might play an important role in adipose tissue.

### 3.2. miR-129-5p Inhibited White Adipogenesis in Noncommitted Multipotent Progenitor Cells In Vitro

**Figure 2:**
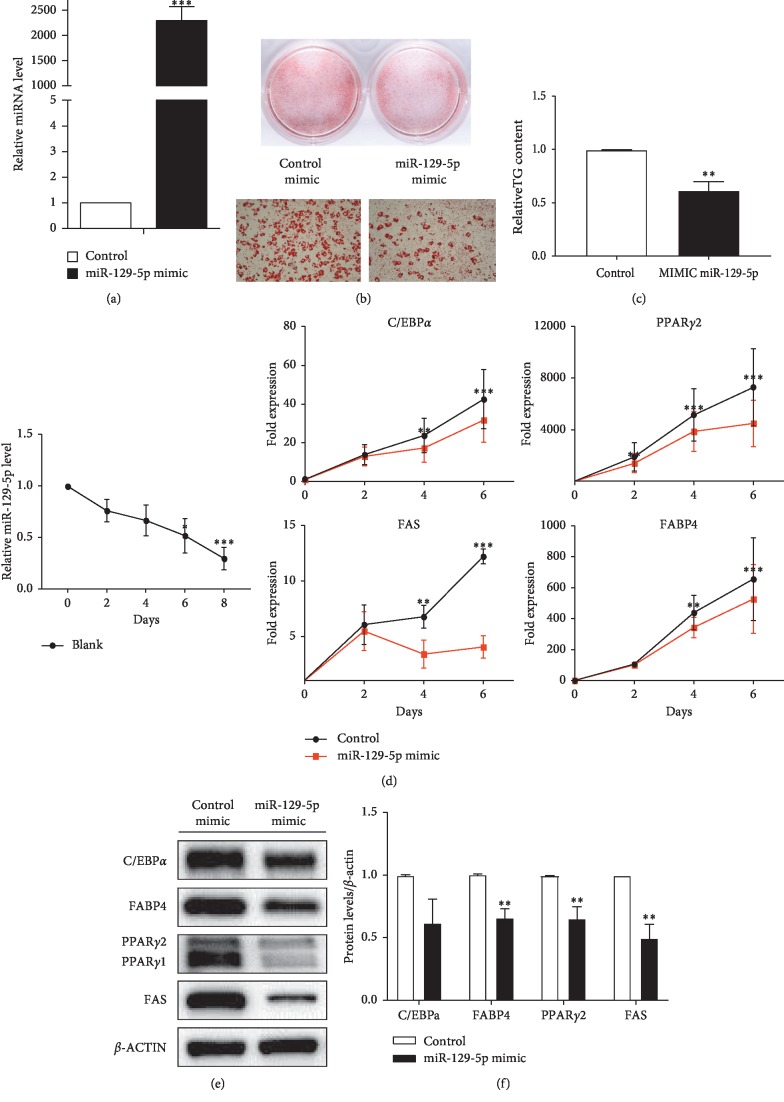
White adipocyte differentiation impaired by microRNA mimic-mediated overexpression of miR-129-5p. (a) Expression levels of miR-129-5p. (b) Oil Red O staining of mature adipocytes. The top two images were captured by a camera; the lower two images were acquired with a microscope at 100x amplification. (c) Relative TG content of cells isolated from EWAT. (d) The time course of miR-129-5p expression during normal adipogenic differentiation was detected, and related white adipogenic genes were qualified by RT-qPCR. (e) Protein levels of the target genes were determined by western blot. (f) Densitometry quantification of western blot. The results of Student's *t*-test are presented as mean ± SEM of a representative of more than three independent experiments (^*∗*^*p* < 0.05, ^*∗∗*^*p* < 0.01, and ^*∗∗∗*^*p* < 0.001).

We first overexpressed miR-129-5p by constructing miR-129-5p mimics. A negative control and miR-129-5p mimics were transfected in stromal vascular fraction (SVF) from subcutaneous fat tissues of male mice before 100% confluency. Subsequently, the cells were induced to differentiate into white mature adipocytes according to standard differentiation protocol (see Section 2). The expression of miR-129-5p was obviously enhanced after transduced miR-129-5p mimics (Figure 2(a)). The results of Oil Red staining and TG determination (Figures 2(b) and 2(c)) showed that the differentiation was inhibited by miR-129-5p mimics. The cells were harvested at the indicated times. The mRNA expression of key genes involved in adipogenisis, including C/EBP*α* and PPAR*γ*, were detected (Figure 2(d)). Consistently, the protein expression of FABP4, PPAR*γ*, and FAS was significantly inhibited by the expression of miR-129-5p (Figures 2(e) and 2(f)). This finding indicated that SVF from subcutaneous white fat tissues transfected with miR-129-5p mimics had a lower differentiation capacity.

### 3.3. miR-129-5p Also Inhibited Beige and Brown Adipogenesis in Cells of SVF In Vitro

**Figure 3:**
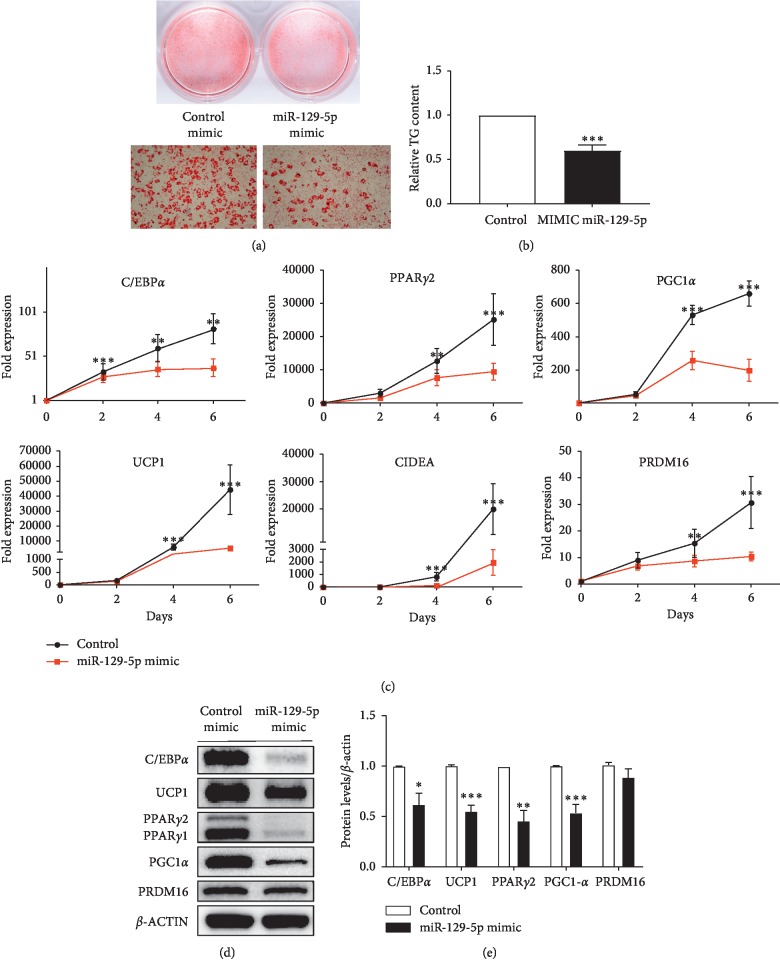
miR-129-5p mimics inhibited browning of SVF from abdominally subcutaneous fat tissues in male mice. SVF from abdominally subcutaneous fat tissues was induced to differentiate into brown adipocytes. (a) Oil Red O staining of mature beige adipocytes. (b) Relative TG content of these cells. (c) The time course of miR-129-5p expression during normal beige adipogenic differentiation and C/EBP*α*, PPAR*γ*2, and UCP1 gene expressions were quantified by RT-qPCR. (d) Marker proteins and genes of brown adipocytes were determined by western blot. (e) Densitometry quantification of western blot. Data were analyzed with Student's *t*-test and is presented as mean ± SEM of a representative of more than three independent experiments (^*∗*^*p* < 0.05, ^*∗∗*^*p* < 0.01, and ^*∗∗∗*^*p* < 0.001).

Next, miR-129-5p was also overexpressed in SVF from abdominally subcutaneous fat tissues. Then, the transfected cells were induced to differentiate toward a brown adipocyte through the corresponding protocol (see Section 2), which is called “beige adipogenesis” or “white adipocyte browning.” The results of Oil Red staining and TG determination (Figures 3(a) and 3(b)) indicated that the beige adipogenesis was also inhibited. Additionally, the expression of adipogenic genes and specific markers of brown mature adipocytes such as the protein expression of UCP1, PRDM16, and PPAR*γ* were evidently reduced (Figures 3(c)–3(e)).

**Figure 4:**
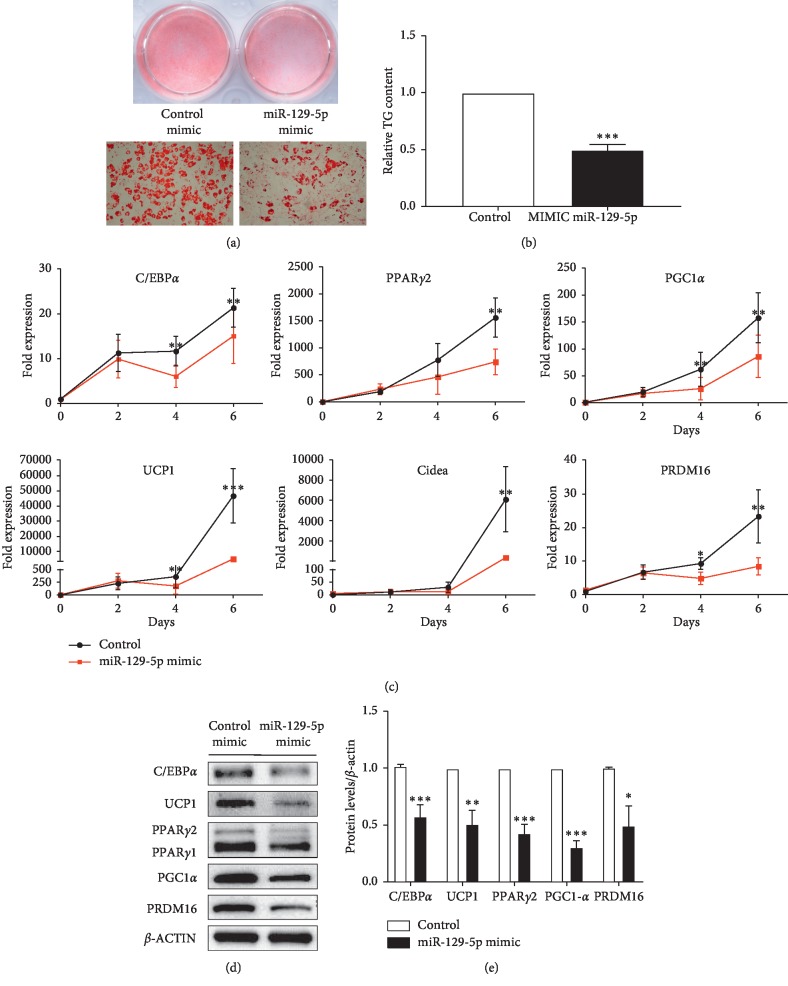
Overexpression of miR-129-5p impaired brown adipocyte differentiation. (a) Oil Red O staining of mature brown adipocytes. (b) Relative TG content of the mature brown adipocytes. (c) The time course of miR-129-5p expression during normal brown adipogenic differentiation and the expression of regulators involved in adipogenesis were analyzed by RT-qPCR. (d) Protein levels of the target genes were determined by western blot. (e) Densitometry quantification of western blot in (d). The SVF from interscapular fat tissues was induced to differentiate toward the brown adipocytes. Data were analyzed with Student's *t*-test and were presented as mean ± SEM of a representative of more than three independent experiments (^*∗*^*p* < 0.05, ^*∗∗*^*p* < 0.01, and ^*∗∗∗*^*p* < 0.001).

Furthermore, we separated SVF cells in the interscapular fat tissues and cultured with the same method as the program of beige adipocyte differentiation. The differentiation was still reduced by miR-129-5p mimics (Figures 4(a) and 4(b)). The markers of adipocyte differentiation and specific markers of brown mature adipocytes were significantly downregulated (Figures 4(c)–4(e)).

### 3.4. miR-129-5p Downregulated Autophagy Pathway

**Figure 5:**
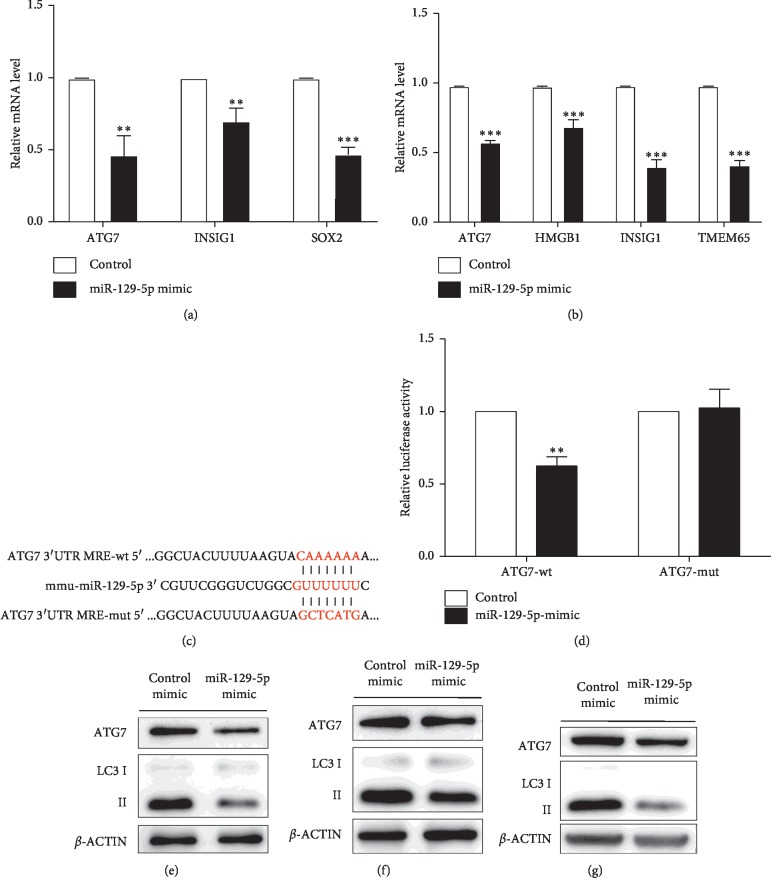
ATG7 was directly downregulated by miR-129-5p in vitro. (a) Predicted target genes were validated by RT-qPCR in SVF white adipocytes. (b) Predicted target genes were validated by RT-qPCR in beige adipocytes. (c) miR-129-5p regulatory element in the 3′UTR of mouse ATG7 was identified by Targetscan. MRE, miRNA regulatory element; MRE-wt, wild-type MRE; MRE-mut, mutated MRE. (d) The effect of miR-129-5p mimics on the reporter construct containing ATG7-3′UTR in MRE-wt or MRE-mut as determined in HEK 293T cells. Relative luciferase units (RLU) are shown. (e–g) ATG7 and LC3I/II were determined by western blot transfected control and miR-129-5p mimics in mature white, beige, and brown adipocytes from SVF. Error bars represent SEM of more than three independent experiments (^*∗∗*^*p* < 0.01 and ^*∗∗∗*^*p* < 0.001).

TargetScan and miRDB were used to identify candidate targets of miR-129-5p. RT-qPCR was then employed to detect related candidates. The results identified ATG7, INSIS1, and SOX2 as candidate genes in mature white adipocytes (Figure 5(a)). ATG7, HMGB1, INSIS1, and TMEM65 were also selected in the mature beige fat cells (Figure 5(b)).

To further confirm whether miR-129-5p directly targeted these candidate genes, a dual luminescence assay was executed in HEK 293T cells. Firstly, reporter constructs containing luciferase cDNA linked to 3′ UTR sequences of candidate mRNAs including wild type and the mutant were generated, such as the specific 3′UTR bind site of ATG7 to miR-129-5p (Figure 5(c)). Next, the miR-129-5p mimics or negative control and reporter constructs were cotransfected into HEK 293T cells. The results showed that only ATG7 (an essential autophagy gene) expression was significantly suppressed by miR-129-5p (Figure 5(d)).

Next, we observed the autophagic flux in mature white, beige, and brown adipocytes through determining the LC3I/II and ATG7 protein expression by western blot after overexpressing and inhibiting miR-129-5p. The results showed that overexpression of miR-129-5p could inhibit autophagy (Figure 5(e)–5(g)), while autophagy was prompted when inhibiting miR-129-5p (Supplementary Figures [Supplementary-material supplementary-material-1]–[Supplementary-material supplementary-material-1]).

### 3.5. Serum miR-129-5p Is Positively Correlated with Obesity in Humans

**Figure 6:**
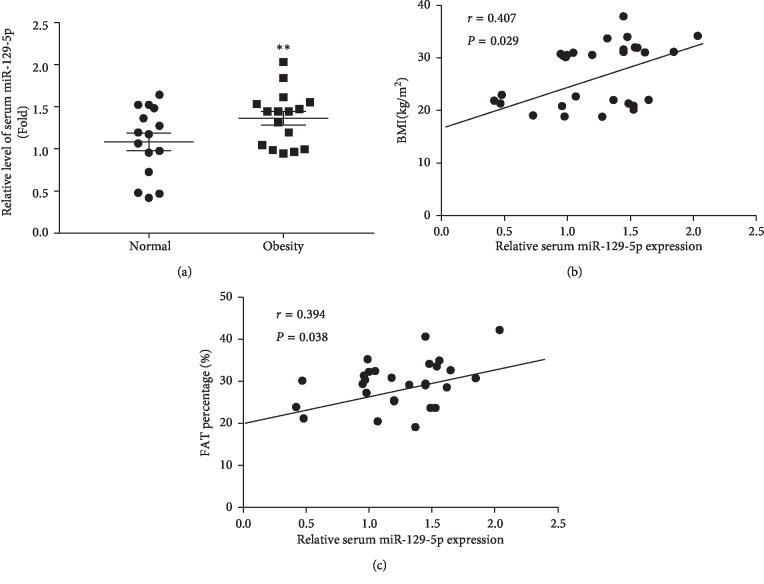
The association of serum miR-129-5p with obesity in humans. (a) Relative miR-129-5p expression level was verified by RT-qPCR in the serum samples from the normal weight group (*n* = 15) and patients with simple obesity (*n* = 16); ^*∗∗*^*p* < 0.01 compared with normal weight subjects. (b) Circulating level of miR-129-5p was detected to positively correlate with BMI in humans; *n* = 31, *r* = 0.407, and *p* < 0.05. (c) Serum miR-129-5p was found to be associated with fat percentage in participants; *n* = 31, *r* = 0.394, and *p* < 0.05.

To confirm whether miR-129-5p plays an important role in humans with obesity, we detected the serum level of miR-129-5p in 15 normal weight participants and 16 patients with simple obesity and analyzed the correlation between serum miR-129-5p with obesity indices. Anthropometric characteristics and biochemical measurements of study participants are summarized in Supp. [Supplementary-material supplementary-material-1]. As we expected, the miR-129-5p level was elevated in patients with simple obesity compared with the normal weight subjects (Figure 6(a); *p* < 0.01). Interestingly, a positive correlation between miR-129-5p level and obesity indices was evident, including BMI (Figure 6(b); *n* = 31, *r* = 0.407, and *p* < 0.029) and fat percentage (Figure 6(c); *n* = 31, *r* = 0.394, *p* < 0.038), suggesting a link between obesity and miR-129-5p level. Taken together, these results demonstrated that circulating miR-129-5p might be a biomarker of obesity.

## 4. Discussion

This study showed that the content of miR-129-5p was increased in the white adipose tissue (WAT) of the mouse model with obesity and participated in adipogenesis. Overexpression of miR-129-5p inhibited adipogenesis in the SVF of white adipose tissue in vitro. miR-129-5p mimics decreased the content of lipid droplet and the expression of the key regulators of adipocyte differentiation, FABP4, C/EBP, PPAR*γ*, etc. Furthermore, when inhibiting the expression of miR-129-5p, the process of white adipogenic differentiation is prompted (Supplementary [Supplementary-material supplementary-material-1]). The regulating effect of miRNAs on obesity always functions by mediating adipogenesis and lipid accumulation, and they can target fat tissues distributed in different parts of the body [12]. As previously reported, miR-128-3P could directly target PPAR*γ* to suppress the 3T3-L1 preadipocyte differentiation and bind with SERTAD2 to drive triglyceride hydrolysis and lipolysis [20]. Gm15290 was one of the most upregulated lncRNAs in the adipocytes of *ob/ob* mice sponged miR-27b identified as a PPAR*γ* targeting miRNA to positively regulate adipogenesis [21].

Although the amount of brown adipose tissue (BAT) in human adults had been previously thought to be minimal, recent studies demonstrated that adult humans have substantial amounts of functioning BAT [22–24]. Loss of BAT activity may contribute to obesity and development of insulin resistance. We observed that miR-129-5p inhibited the brown adipocyte differentiation in scapular adipose tissues in vitro. Also, we observed that miR-129-5p could block the differentiation of SVF from EWAT into BAT. miR-129-5p mimics decreased the expression of the specific and key genes involved in brown adipocytes differentiation and function, UCP1, CIDEA, PRDM16, etc. While inhibiting miR-129-5p, more mature beige and brown adipocytes were observed compared with negative control inhibitors (Supplementary Figures [Supplementary-material supplementary-material-1] and [Supplementary-material supplementary-material-1]). These results indicate that miR-129-5p may be an essential regulator of brown fat adipogenesis and further imply that there is a novel mechanism governing BAT activation and WAT browning.

Autophagy is essential for adipocyte differentiation, and ATG5 or ATG7 knockdown inhibits differentiation of 3T3-L1 preadipocyte [25]. Researchers have analyzed autophagy-related genes during adipocyte differentiation using publicly accessible data, which showed that autophagy may upregulate key pathways related to adipocyte differentiation including the mTOR, Jak-STAT, insulin, and adipocytokine signaling pathways [26]. It was reported that ATG7 deletion in 3T3-L1 preadipocyte inhibited cell differentiation [27]. Under the special conditions like starvation, autophagy could remove ubiquitinated AMPK which could block the mTOR signal pathway to allow the accelerated fat accumulation and the process of differentiation [25]. ATG7-mediated autophagy has been confirmed to play an important role in normal adipogenesis and inhibition of autophagy by disrupting ATG7 leading to less white adipose tissues, and the WAT contains more mitochondria [27]. miR-129-5p may directly regulate ATG7 and reduce white adipogenic differentiation. Endoplasmic reticulum (ER) stress inhibits autophagic flux by blocking autophagosome-lysosome fusion in trophoblast cells [28]. ER-phagy helps to ameliorate the effect of ER stress through the degradation of ER membranes [29]. Nrf1, an ER-localized transcription factor, was identified as a critical driver in the process of adaptive increase of proteasomal activity, which is indispensable to brown adipose tissue (BAT) thermogenic function. Brown adipocyte-specific deletion of Nrf1 results in ER stress. Our experiments also showed that, based on these research studies, we speculated that miR-129-5p may block autophagic flux to disrupt ER homeostasis to diminish mitochondrial function of beige and brown adipocytes [30]. Although it is possible that miR-129-5p mediates the inhibition of adipocyte differentiation through ATG7-related autophagy pathway, the detailed mechanisms still need to be investigated further.

Recent studies have revealed that miRNAs can be treated as biomarkers in metabolic diseases [31], B-cell lymphomagenesis [32], age-related disease [33], and various cancers [34]. This study identified that miR-129-5p was elevated in human serum with obesity and positively correlated with obesity indices, such as BMI and fat percentage.

## 5. Conclusion

Our results have revealed the biological function of miR-129-5p for the first time in regulating adipocyte differentiation. The present research also demonstrated that serum miR-129-5p could be a potential biomarker for obesity in human, which will provide identification of more therapeutic targets and strategies against obesity and related metabolic disorders.

## Figures and Tables

**Table 1:** Primer sequences employed in this study.

Gene name	Forward primer sequence (5′–3′)	Reverse primer sequence (5′–3′)
C/EBP*α*	CAAGAACAGCAACGAGTACCG	GTCACTGGTCAACTCCAGCAC
PPAR*γ*	TCGCTGATGCACTGCCTATG	GAGAGGTCCACAGAGCTGATT
UCP1	AGGCTTCCAGTACCATTAGGT	CTGAGTGAGGCAAAGCTGATTT
CIDEA	TGCTCTTCTGTATCGCCCAGT	GCCGTGTTAAGGAATCTGCTG
PRDM16	CCACCAGCGACTTCAC	GCAGGACTCTCGTAGCTCGAA
FABP4	AGCATCATAACCCTAGATGGCG	CATAACACATTCCACCACCAGC
ATG7	GTTCGCCCCCTTTAATAGTGC	TGAACTCCAACGTCAAGCGG
SOX2	GCGGAGTGGAAACTTTTGTCC	CGGGAAGCGTGTACTTATCCTT
HMGB1	GGCGAGCATCCTGGCTTATC	GGCTGCTTGTCATCTGCTG
TMEM65	CCATCGCACAAGGTAAGCG	GACAGGGGTCTGAGAAGTAGG
INSIG1	CACGACCACGTCTGGAACTAT	TGAGAAGAGCACTAGGCTCCG
*β*-ACTIN	GCCAGCCTCTCCTGATTTTAGTGT	GGGAACACAAAAGACCTCTTCTGG

**Table 2:** Clinical characteristics of validation study participants by weight category.

Variable	Normal weight control (mean ± SD)	Obese patients (mean ± SD)	*p* value
*Demographic data*			
Sex (male) (*n* (%))	15 (100%)	16 (100%)	
Age (years)	27.22 ± 1.77	27.45 ± 1.79	0.696

*Anthropometric measurements*			
BMI (kg/m^2^)	21.19 ± 1.35	32.01 ± 1.91	**0.0001**
WC (cm)	76.57 ± 7.89	105.96 ± 6.17	**0.0001**
HC (cm)	93.14 ± 5.02	110.37 ± 5.26	**0.0001**
WHR	0.82 ± 0.06	0.96 ± 0.04	**0.0001**
Fat percentage (%)	25.07 ± 4.89	32.64 ± 4.09	**0.0001**

*Biochemical characteristics*			
Glu (mmol/L)	5.07 ± 3.15	5.35 ± 3.65	**0.016**
HBA1C (%)	5.13 ± 0.20	5.43 ± 0.43	**0.0001**
TC (mmol/L)	4.67 ± 0.83	5.83 ± 1.29	**0.002**
TG (mmol/L)	0.92 ± 0.38	2.71 ± 1.97	**0.001**
HDL (mmol/L)	1.54 ± 0.29	1.13 ± 0.21	**0.0001**
LDL (mmol/L)	2.69 ± 0.64	3.67 ± 1.00	**0.001**
Insulin (IU/L)	6.42 ± 2.64	19.37 ± 8.48	**0.0001**

The data are presented as mean ± SD, and the *p* value was calculated using a two-tailed test; significant *p* values are indicated in bold. BMI, body mass index; WC, waist circumference; HP, hip circumference; WHR, the rate of WC to HP; Glu, glucose; HbAlc, glycosylated hemoglobin; TC, total cholesterol; TG, triglyceride; HDL, high-density lipoproteins; LDL, low-density lipoproteins.

## Data Availability

The data used to support the findings of this study are available from the corresponding author upon request.

## Supplementary Materials

Supplementary Materials Supplementary Table 1: the sequences for the miR-129-5p mimic and inhibitor. Supplementary Figure 1: white adipocyte differentiation prompted by microRNA inhibitor-mediated low expression of miR-129-5p. Supplementary Figure 2: miR-129-5p inhibitors prompted browning of SVF from abdominally subcutaneous fat tissues in male mice. Supplementary Figure 3: reduction of miR-129-5p prompted brown adipocyte differentiation. Supplementary Figure 4: miR-129-5p level was detected in EWAT from different obese mouse models. Supplementary Figure 5: (a) HMGB1 and TMEM65 were detected by RT-qPCR in SVF white adipocytes. (b) Predicted target genes SOX2 were detected by RT-qPCR in beige adipocytes. (The difference was not statistically significant.) Supplementary Figure 6: (a–c) the time course of ATG7 expression 47 during white, beige, and brown adipogenic differentiation when transfected control and miR-129-5p mimics were quantified by RT-qPCR. Supplementary Figure 7: (a–c) ATG7 and LC3I/II were determined by western blot transfected control and miR-129-5p inhibitors in mature white, beige, and brown adipocytes from SVF. Supplementary Figure 8: (a–f) all uncropped western bolt bands.
