# Supplementary material for: miR-129-5p Inhibits Adipogenesis through Autophagy and May Be a Potential Biomarker for Obesity
Source: Int J Endocrinol. 2019 Nov 6;2019:5069578. doi: 10.1155/2019/5069578 (PMC6875017; doi:10.1155/2019/5069578)
Supplement: Supplementary Materials — Supplementary Table 1: the sequences for the miR-129-5p mimic and inhibitor. Supplementary Figure 1: white adipocyte differentiation prompted by microRNA inhibitor-mediated low expression of miR-129-5p. Supplementary Figure 2: miR-129-5p inhibitors prompted browning of SVF from abdominally subcutaneous fat tissues in male mice. Supplementary Figure 3: reduction of miR-129-5p prompted brown adipocyte differentiation. Supplementary Figure 4: miR-129-5p level was detected in EWAT from different obese mouse models. Supplementary Figure 5: (a) HMGB1 and TMEM65 were detected by RT-qPCR in SVF white adipocytes. (b) Predicted target genes SOX2 were detected by RT-qPCR in beige adipocytes. (The difference was not statistically significant.) Supplementary Figure 6: (a–c) the time course of ATG7 expression 47 during white, beige, and brown adipogenic differentiation when transfected control and miR-129-5p mimics were quantified by RT-qPCR. Supplementary Figure 7: (a–c) ATG7 and LC3I/II were determined by western blot transfected control and miR-129-5p inhibitors in mature white, beige, and brown adipocytes from SVF. Supplementary Figure 8: (a–f) all uncropped western bolt bands. [file 5069578.f1.pdf]

Supplementary materials

Table legends

Supplementary table 1: the sequences for miR-129-5p mimic and inhibitor.

Product name	Sense(5'-3')	antisense (5'-3')
micrONTM mimic Negative Control #22	UUUGUACUACACAAAAG UACUG	CAGUACUUUUGUGUAG UACAAA
micrONTM mmu-miR-129-5p mimic	CUUUUUGCGGUCUGGGC UUGC	GCAAGCCCAGACCGCAA AAAG
micrOFFTM inhibitor Negative Control #22	CAGUACUUUUGUGUAG UACAAA	/
micrOFFTM mmu-miR-129-5p inhibitor	GCAAGCCCAGACCGCAA AAAG	/

Figure legends

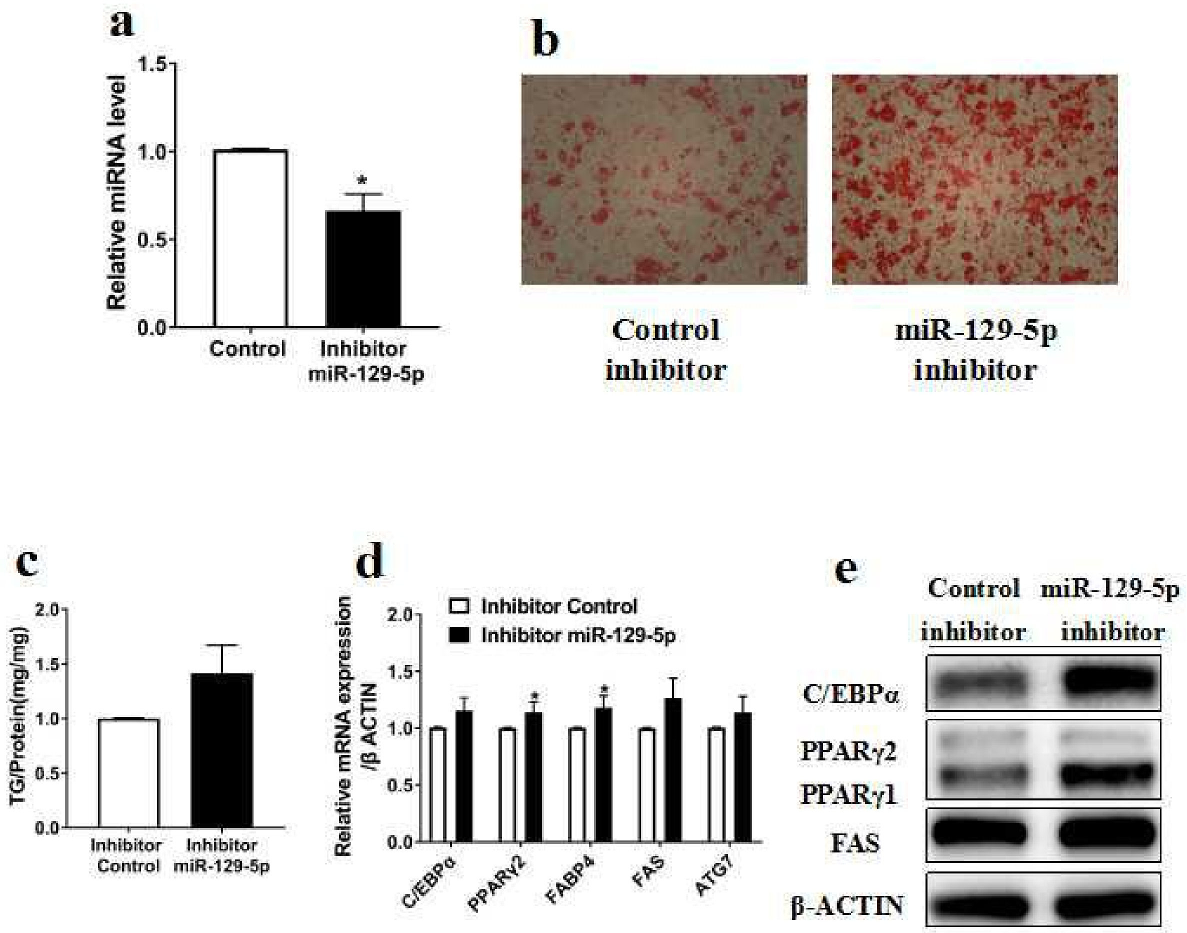

Supplementary material figure1: White adipocyte differentiation prompted by microRNA-inhibitor-mediated low-expression of miR-129-5p. (a) Expression levels of miR-129-5p. (b) Oil Red O staining of mature adipocytes. These images were acquired with a microscope at 100x amplification. (c) Relative TG content of cells isolated from EWAT. (d) The relative white adipogenic genes and ATG7 were qualified by RT-qPCR. (e) Protein levels of the target genes were determined by western blot. Student's t-test presented as mean±SEM of a representative of more than three independent experiments (*$P < 0.05$).

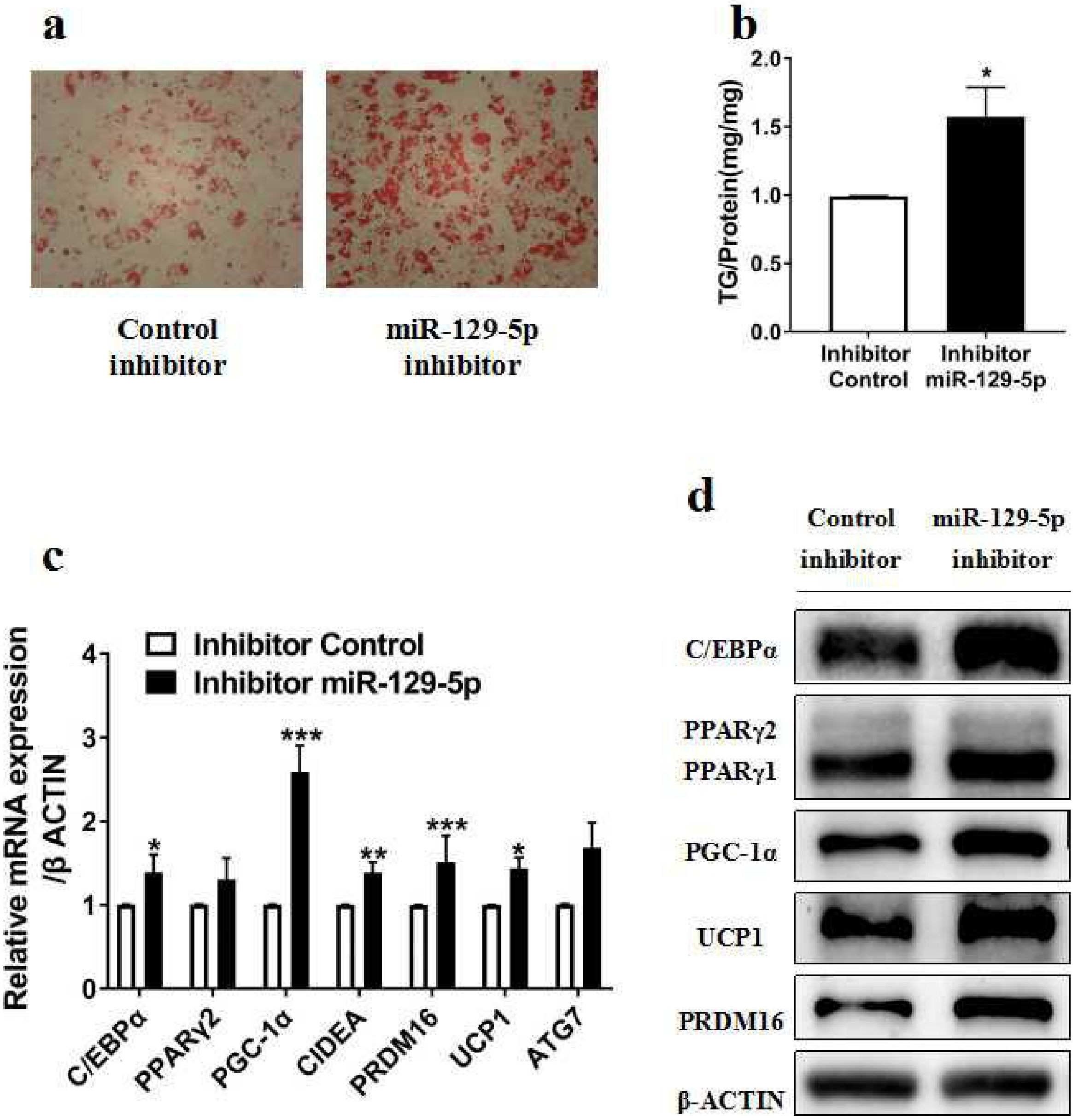

Supplementary material figure 2: miR-129-5p inhibitors prompted browning of SVF from abdominally subcutaneous fat tissues in male mice. SVF from abdominally subcutaneous fat tissues were induced to differentiate into brown adipocytes. (a) Oil Red O staining of mature beige adipocytes at 100x amplification. (b) Relative TG content of these cells. (c) C/EBPα, PPARγ2, UCP1 and ATG7 gene expressions were quantified by RT-qPCR. (d) Marker proteins and genes of brown adipocyte were determined by western blot. Data was analyzed with Student's t-test and is presented as mean±SEM of a representative of more than three independent experiments (*$P < 0.05$, **$P < 0.01$, and ***$P < 0.001$).

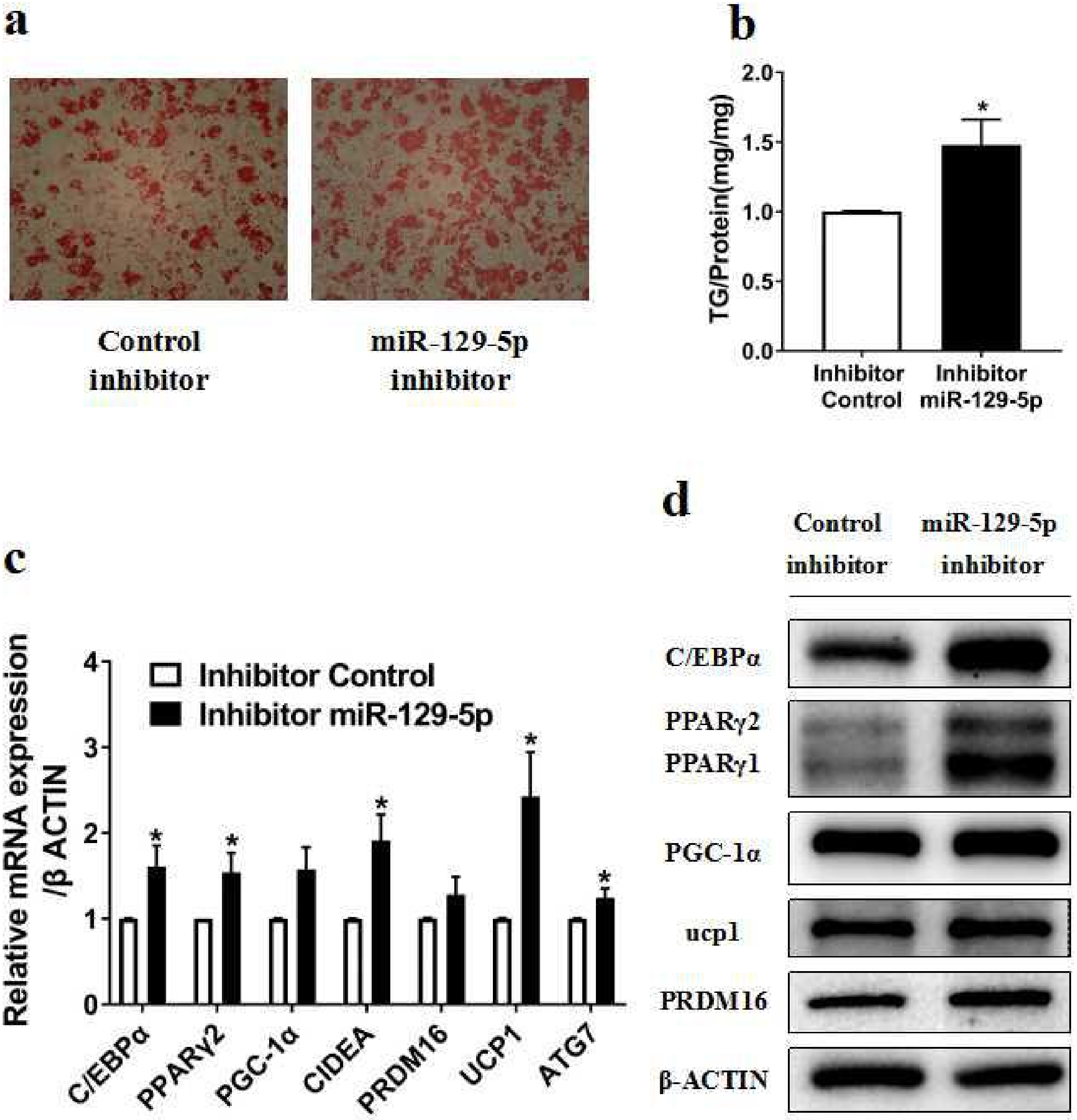

Supplementary material figure3: Reduction of miR-129-5p prompted brown adipocyte differentiation. (a) Oil Red O staining of mature brown adipocytes. (b) Relative TG content of the mature brown adipocytes. (c) The expression of regulators involved in adipogenesis and ATG7 were analyzed by RT-qPCR. (d) Protein levels of the target genes were determined by western blot. The SVF from interscapular fat tissues were induced to differentiate toward the brown adipocytes. Data was analyzed with Student's t-test and was presented as mean±SEM of a representative of more than three independent experiments (*$P < 0.05$).

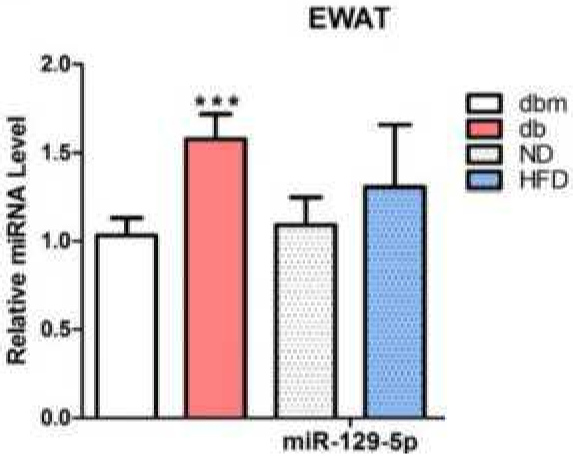

Supplementary material figure4: miR-129-5p level was detected in EWAT from different obese mouse models. (a).Relative expression level of miR-129-5p in epididymal white adipose tissue (EWAT) of *db/db* mice, high-fat diet(HFD) mice compared with wild-type mice (WT), normal feeding mice(ND) n=10, *p＜0.05, ***p<0.001.

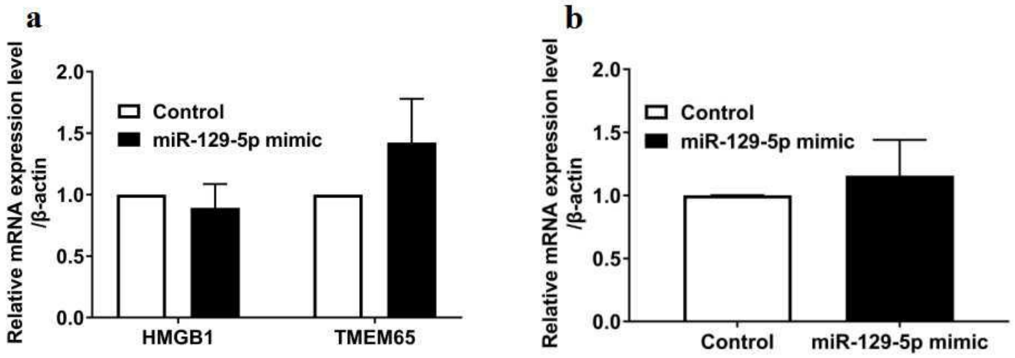

Supplementary materials figure 5: (a) HMGB1 and TMEM65 were detected by RT-qPCR in SVF white adipocytes. (b) Predicted target genes SOX2 were detected by RT-qPCR in beige adipocytes.(The difference was not statistically significant.)

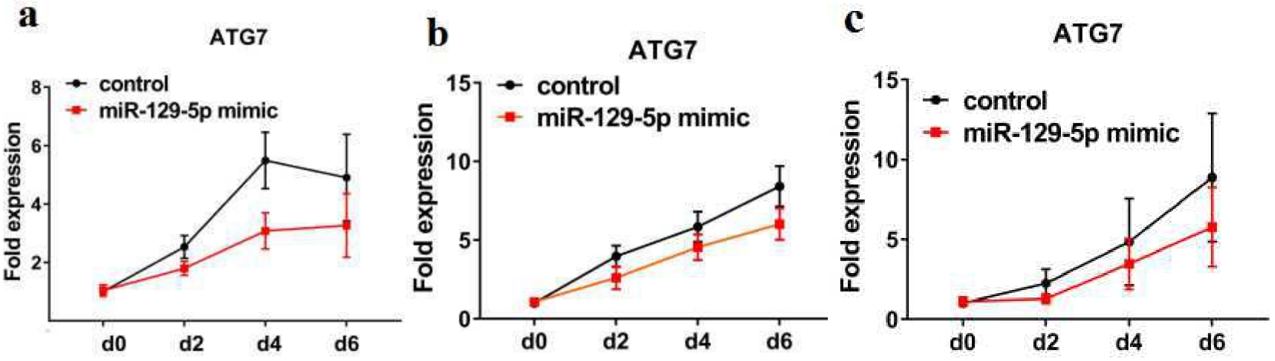

Supplementary materials figure 6: (a-c) The time course of ATG7 expression during white,beige and brown adipogenic differentiation when transfected control and miR-129-5p mimics were quantified by RT-qPCR.

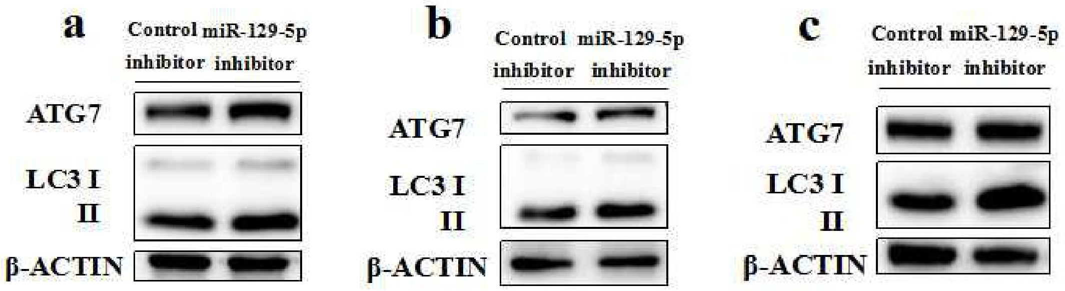

Supplementary materials figure7: (a-c) ATG7 and LC3I/II were determined by western blot transfected control and miR-129-5p inhibitors in mature white, beige and brown adipocytes from SVF.

a **White adipocyte from SVF**

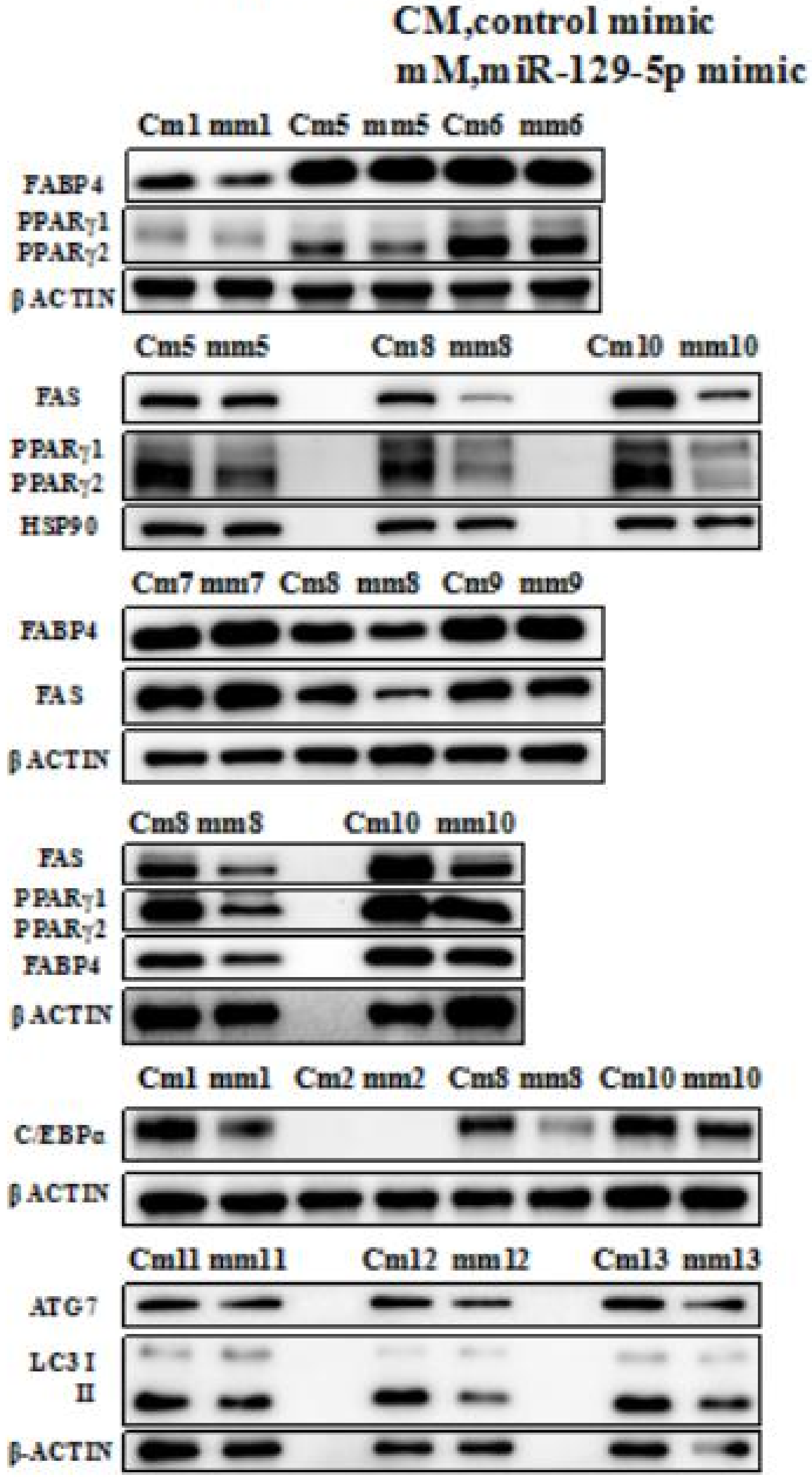

b Beige adipocyte from SVF

CM,control mimic
mM,miR-129-5p mimic

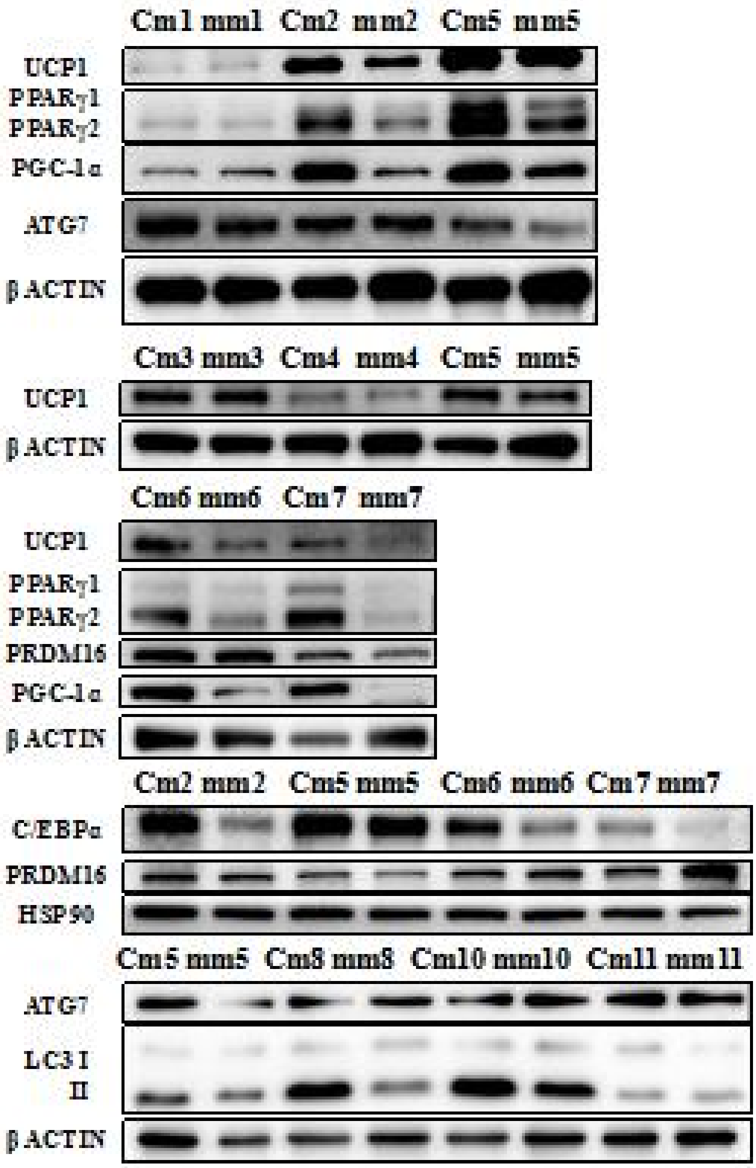

c Brown adipocyte from SVF

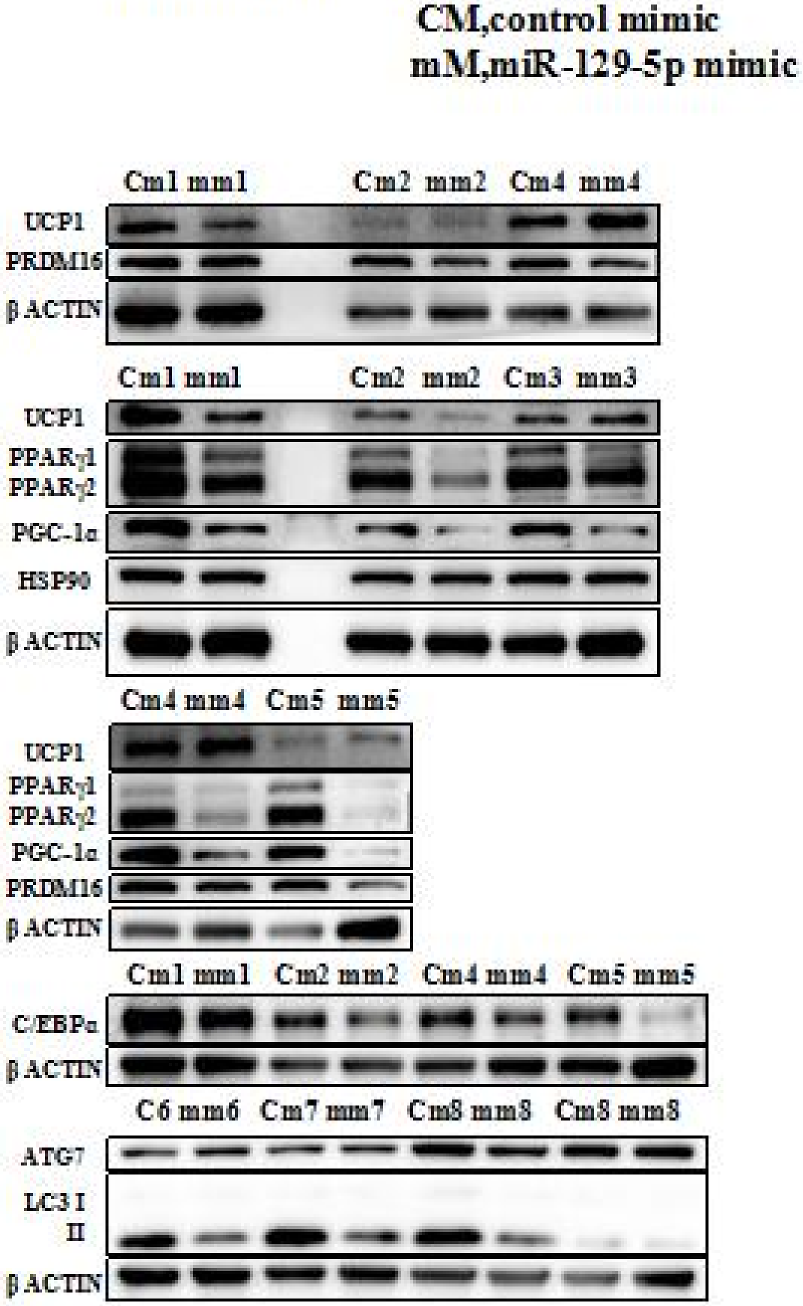

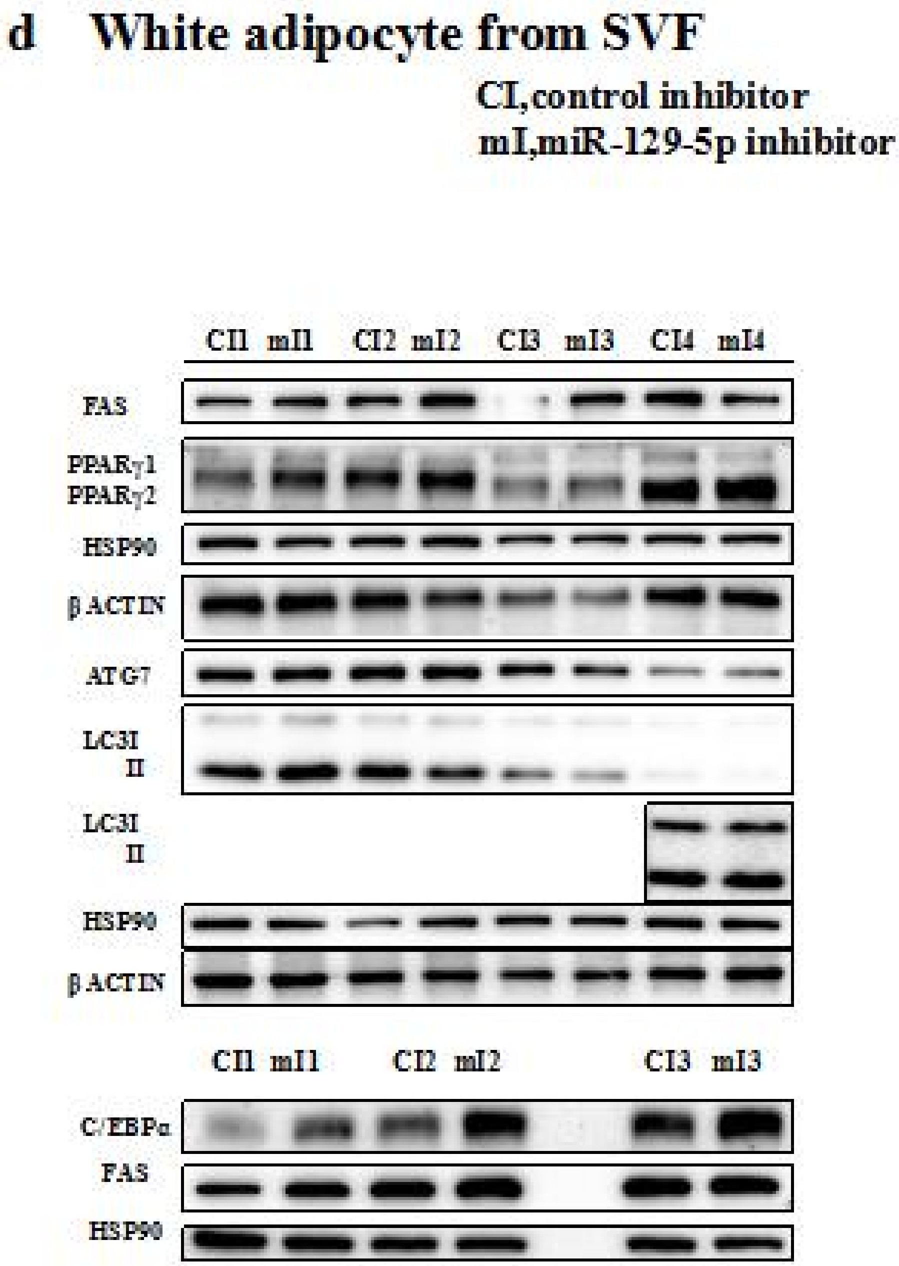
d White adipocyte from SVF
CI,control inhibitor
mI,miR-129-5p inhibitor
CI1 mI1 CI2 mI2 CI3 mI3 CI4 mI4
FAS
PPARγ1
PPARγ2
HSP90
β ACTIN
ATG7
LC3I
II
LC3I
II
HSP90
β ACTIN
CI1 mI1 CI2 mI2 CI3 mI3
C/EBPα
FAS
HSP90

e Beige adipocyte from SVF

CI,control inhibitor
mI,miR-129-5p inhibitor

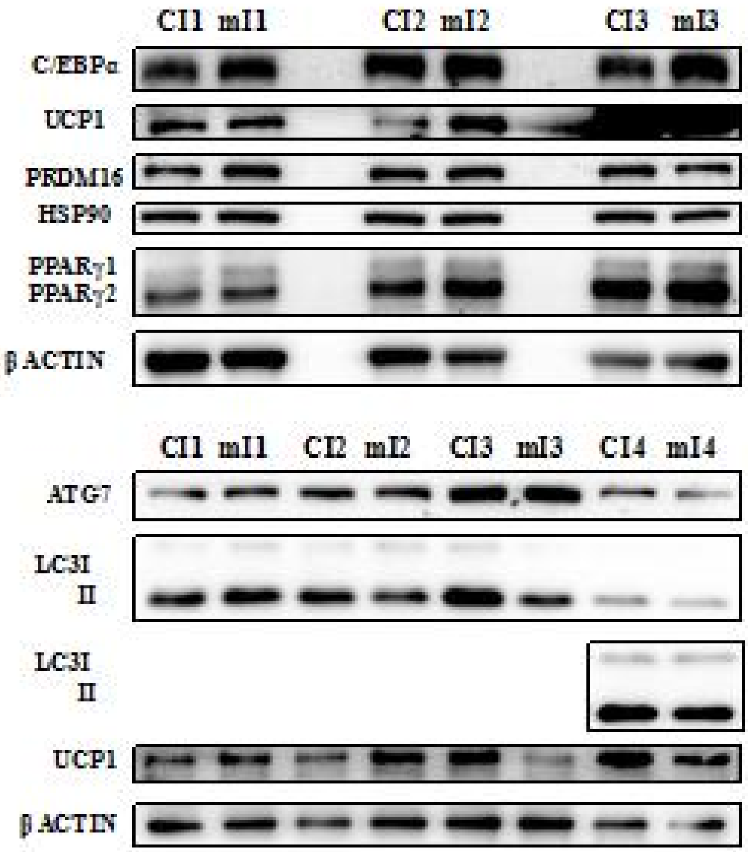

f Brown adipocyte from SVF

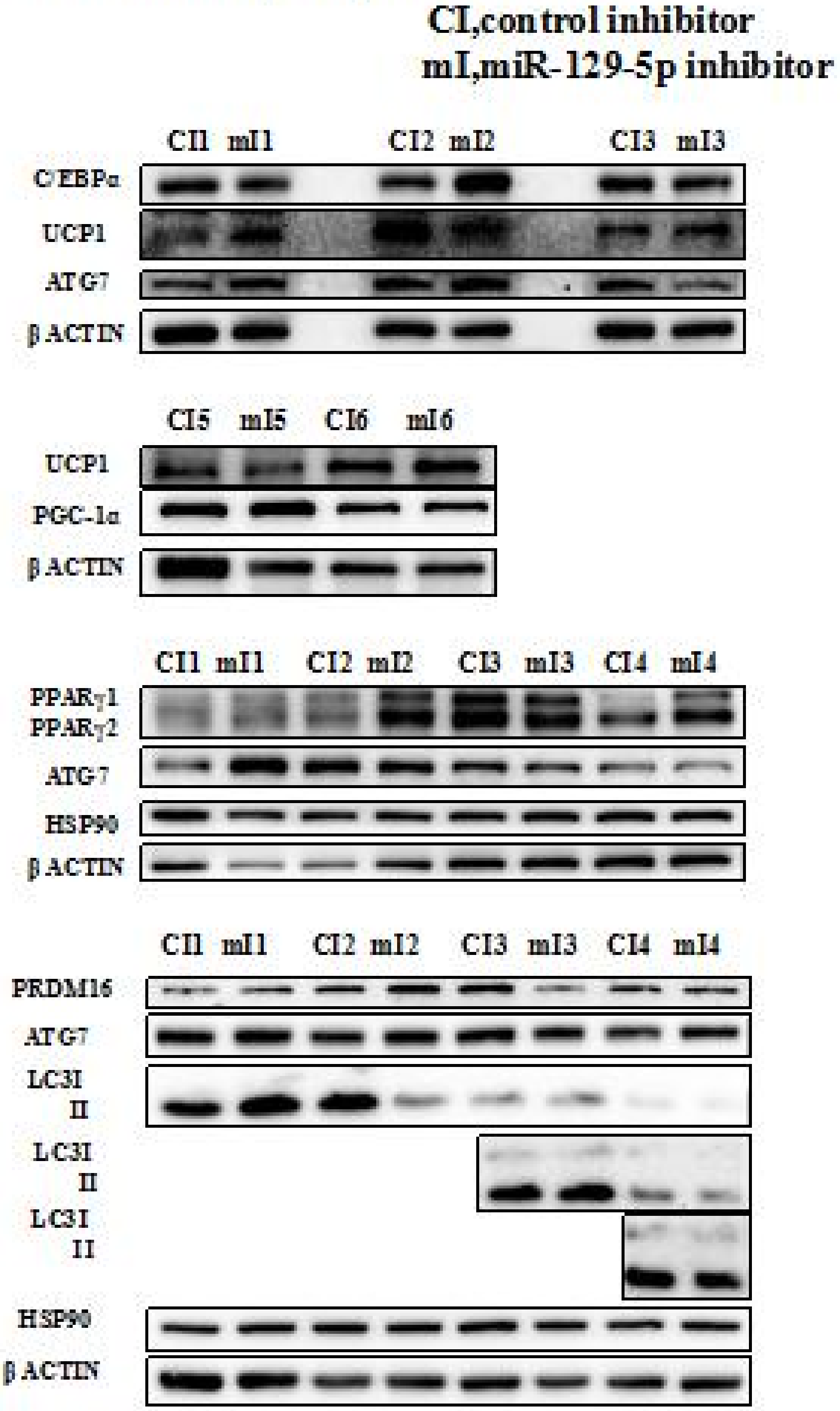

66

67 **Supplementary figure 8: (a-f)** All uncropped western bolt bands.
